# Assessing the Efficacy of Mdm2/Mdm4-Inhibiting Stapled Peptides Using Cellular Thermal Shift Assays

**DOI:** 10.1038/srep12116

**Published:** 2015-07-10

**Authors:** Ban Xiong Tan, Christopher J. Brown, Fernando J. Ferrer, Tsz Ying Yuen, Soo Tng Quah, Boon Hong Chan, Anna E. Jansson, Hsiang Ling Teo, Pär Nordlund, David P. Lane

**Affiliations:** 1 p53 Laboratory, A*STAR, Singapore; 2 Institute of Chemical and Engineering Sciences, A*STAR; 3 School of Biological Sciences, Nanyang Technological University, Singapore

## Abstract

Previous publications on stapled peptide inhibitors against Mdm2/Mdm4-p53 interactions have established that this new class of drugs have the potential to be easily optimised to attain high binding affinity and specificity, but the mechanisms controlling their cellular uptake and target engagement remain elusive and controversial. To aid in understanding the rules of peptide and staple design, and to enable rapid optimisation, we employed the newly-developed cellular thermal shift assay (CETSA). CETSA was able to validate stapled peptide binding to Mdm2 and Mdm4, and the method was also used to determine the extent of cellular uptake, cellular availability, and intracellular binding of the endogenous target proteins in its native environment. Our data suggest that while the stapled peptides engage their targets intracellularly, more work is needed to improve their cellular entry and target engagement efficiency *in vivo*. CETSA now provides a valuable tool to optimize such *in vivo* properties of stapled peptides.

Commonly dubbed the ‘guardian of the genome’, the tumour suppressor *p53* has been found to be mutated in half of all cancers^1^,^2^, and with more than 70 000 publications describing the gene, it is undoubtedly one of the most important and well-studied cancer-related genes. About 50% of cancers lack p53 activity due to mutations within the coding regions while the other 50% have aberrations that, in one way or another, reduce the function of the wild type p53 pathway^3^. In either situation, the transcriptional role of p53 is perturbed, resulting in the inability to activate or repress p53 target genes that govern cell cycle arrest or apoptosis. In additional, transcription-independent roles of p53 are disrupted. Ultimately, the lack of p53 activity results in unfettered cell division, genomic instability and cellular transformation.

Wild type p53 can be reactivated by inhibiting its structurally similar negative regulators Mdm2 and Mdm4. Blocking the interactions between p53 and Mdm2/Mdm4 prevents the occlusion of the p53 transactivation domain, as well as preventing p53 nuclear export, ubiquitination and proteasomal degradation^4^,^5^. This results in the accumulation of p53, allowing both p53 transcription-dependent and independent activity, fulfilling its role as a tumour suppressor. Small molecules, like nutlin-3^6^ or MI-219^7^, have been developed that mimic the p53 N-terminal region that interfaces with Mdm2 (reviewed in Brown *et al.* 2009^8^). More recently, the Mdm2 binding motif of p53 has been used to engineer peptides that bind to Mdm2 and Mdm4^9^,^10^,^11^. To maintain peptide helicity and to allow additional contacts with Mdm2/Mdm4, hydrocarbon staples have been added, increasing the affinity and activity of some of these stapled peptides^12^. Furthermore, hydrocarbon stapling vastly improves the helical stability of the peptides, promotes their cellular uptake and protects them from protease degradation.

The therapeutic effects of modern oncology drugs are obtained by the specific interaction of drugs with their molecular targets, often simply described as “targeted therapy” to distinguish these approaches from more generic cytotoxic drugs and radiation therapy. Therefore, in drug design and optimisation, the knowledge of how well a drug binds to its target is of utmost importance in determining drug efficacy and understanding potentially deleterious side effects. There are several methods to assay for drug-target engagement, and one of the most direct methods is through the use of thermal shift assays^13^,^14^,^15^,^16^. These assays utilise the principle that protein conformation is stabilised by interactions with ligands and the proteins therefore undergo denaturation at higher temperatures. The amount of thermal shift in the presence of ligands correlates well with ligand affinity^17^, and can be used to approximate binding affinities between drugs and their target proteins. Traditionally, these assays require the use of purified proteins, representing a challenge in the case of proteins that are difficult to isolate. Recently, cellular thermal shift assays have been developed to overcome this requirement, using crude cell lysates or even intact cells and tissues rather than purified proteins^18^. Instead of fluorescent dyes that bind to hydrophobic protein cores, thermal denaturation is measured by separating the soluble proteins with centrifugation, followed by Western blotting to quantitate the protein of interest. The added benefit is that CETSA allows the observation of thermal shifts of proteins in their cellular environments, which is more representative of the true target environment, taking account of the presence of other cellular proteins, lipids and biomolecules at high concentrations. CETSA can be also used to assay for drug entry and target engagement within cells^18^.

There is a demand in establishing rapid and reliable assays to deconvolute the complexity of targeting intracellular protein-protein interactions in therapeutics^19^. With increasing evidence that targeting the Mdm2/Mdm4/p53 axis is crucial in the treatment of several types of malignancies, the dual inhibition of both Mdm2 and Mdm4 is potentially a key strategy in therapeutics^20^,^21^. In this study, we utilised CETSA to assay for the efficacy of Mdm2/Mdm4 inhibitory stapled peptides, both in terms of Mdm2/Mdm4 engagement in cells and cell lysates, as well as for entry and availability in cells. The stapled peptides were found to induce large thermal shifts in the stability of their target proteins in cell lysates largely in concordance to their affinities estimated using other techniques with purified proteins, though discrepancies may arise from the use of different approaches to estimate binding affinities. In CETSAs involving whole cells, the thermal shifts were repressed compared to CETSAs involving cell lysates, indicating the poorer efficiency of the stapled peptides in reaching their targets within cells. This study provided evidence that CETSA is an immensely useful tool for drug design and optimisation, not just in target affinity, but also in cell entry and availability and is capable of dealing with novel drug classes such as larger peptide molecules.

## Materials and Methods

### Peptide synthesis and purification

#### Materials

Ramage Chemmatrix resin was obtained from PCAS-Biomatrix (Quebec, Canada). L-amino acids were obtained from Advanced Chemtech (Louisville, KY). Fmoc-threonine, serine, glutamic acid and tyrosine were t-butyl protected and Fmoc-tryptophan was used without BOC protection. Unnatural alkenyl amino acids, (S)-N-Fmoc-2-(4’-pentenyl)alanine and (R)-N-Fmoc-2-(7’-octenyl)alanine denoted as Xs and Xr respectively on the peptide sequences were purchased from OKeanos (Beijing, China). 5-FAM was obtained from GL Biochem (Shanghai, China). All other solvents and reagents were obtained from Sigma-Aldrich. 1, 2-dichloroethane (DCE) was dried overnight over activated molecular sieves and purged with Argon for 30 min prior to use. All other reagents were used as received.

#### Peptide synthesis

The stapled peptides were synthesized manually by Fmoc chemistry at the 0.1 mmol scale using Ramage Chemmatrix resin (0.53 mmol/g). The dry resin was swelled with 1-methyl-2-pyrrolidinone (NMP) before use. The Fmoc protecting group was removed by treatment with 20% piperidine in NMP (15 min). Solutions of the Fmoc-protected amino acids (5 equiv.), diisopropylcarbodiimide (DIC, 5 equiv.) and 1-aza benzotriazole (HOAt, 5 equiv.) in NMP (0.5 M) were activated for 7 min and added to the resin. The coupling time was 1 hour for all amino acids except for (*S*)-N-Fmoc-2-(4’-pentenyl)alanine and (*R*)-N-Fmoc-2-(7’-octenyl)alanine, denoted Xs and Xr, respectively. Those alkenyl amino acids (4 equiv.) were coupled to the peptide resin for two hours. Following deprotection of the final Fmoc group, the peptides were acetylated using a mixture of acetic anhydride/diisopropylethylamine/DMF (2/2/1) for 60 min. After each coupling, deprotection and acetylation reaction, the resin was thoroughly washed with NMP, methanol and diethyl ether.

Ring-closing metathesis of resin-bound, N-acetylated peptides was performed manually using a 5 mg/mL solution of Grubbs I catalyst (20 mol%) in dry 1,2-dichloroethane (DCE) at room temperature under an atmosphere of inert argon (3 × 2 h treatments). After the reaction, the solution was drained, the resin washed with DCE (3 × 1 min), DMSO (1 × 2 h) and MeOH (3 × 1 min) then dried *in vacuo* overnight. Cleavage of the peptide from the resin was achieved using 8 mL of TFA cocktail consisting trifluoroacetic acid/triisopropylsilane/water (95/2.5/2.5) for 2 h followed by filtration and precipitation with diethyl ether. The precipitate was collected by centrifugation, dried and redissolved in a 3:2 mixture of acetonitrile and water.

The 5-FAM was incorporated to the peptide resin after the Fmoc deproctection of the cyclized peptide. 5-FAM (7 equiv) was dissolved in a mixture of DMSO/ DMF 2:1 preactivated with DIC/HOAt (7 equiv) and added to the resin overnight.

The pure peptides (>90% purity) were obtained by purification using a preparative HPLC system (Agilent) on a Jupiter C12 reversed-phase preparative column (Phenomenex, 4 μm, Proteo 90 Å, 250 × 10 mm). The structures of the stapled peptides are depicted in Supplementary Figures 1 and 2.

### Cell culture and drug treatments

OCI/AML-2 cells (DMSZ) were cultured in MEM Alpha supplemented with 20% foetal bovine serum (FBS) and penicillin/streptomycin, in a 37 °C humidified incubator with 5% CO_2_ atmosphere. 24 hours prior to drug treatment, cells were transferred to MEM Alpha containing 10% FBS. Cells were pre-treated with 10 μM MG132 for one hour, before harvesting for cell lysate CETSA or treatment for whole cell CETSA. For whole cell CETSAs, cells were treated with the indicated amounts of nutlin-3 or stapled peptides for four hours before collection. The total amount of DMSO, used to dissolve the compounds, was fixed at 0.35% during incubation.

### T22 p53 β-galactosidase based reporter assay

The T22 p53 β-galactosidase based reporter assay was performed as described by Brown *et al.*^10^. T22 cells, which were stably transfected with a p53 responsive β-galactosidase reporter, were seeded into 96-well plate at a cell density of 8000 cells per well^22^. Cells were maintained in Dulbecco’s Minimal Eagle Medium (DMEM) with 10% FBS and penicillin/streptomycin and incubated for 24 hours before treatment with the stapled peptides for 18 hours. β-galactosidase activity was assayed using the FluoReporter LacZ/Galactosidase Quantitation kit (Invitrogen) as per manufacturer’s instructions. Measurements were made using an Envision plate reader (Perkin Elmer). Experiments were carried out independently thrice.

### Lysate CETSA

Cells pre-treated with 10 μM MG132 for one hour were lysed in kinase buffer (25 mM Tris pH 7.4, 10 mM MgCl2, 2 mM DTT, 1x protease (Roche) and phosphatase inhibitor cocktails (Thermo Scientific)) by dounce homogenisation with a 2 ml Wheaton mortar and pestle. Cell lysates were cleared by centrifugation and aliquoted for treatment with nutlin-3 or stapled peptides. Following a 15 min incubation at room temperature with the drugs, the cell lysates were individually heated to the indicated temperatures for 2 min, cool at room temperature for 2 min, and placed on ice. Insoluble proteins were separated by centrifugation, and the soluble proteins were used for SDS PAGE and Western blotting for the indicated proteins. Three independent replicates were performed.

### Whole cell CETSA

Cells were pre-treated with 10 μM MG132 for one hour before a four hour treatment with the indicated drugs. They were then aliquoted for heating to different temperatures for 2 min, cooled at room temperature for 2 min and placed on ice. Kinase buffer was added to the cells, followed by lysis with three freeze/thaw cycles in liquid nitrogen. Insoluble proteins were separated by centrifugation, and the soluble proteins were used for SDS PAGE and Western blotting. Three independent experiments were performed.

### Confocal microscopy

For confocal microscopy, HCT116 cells were grown in McCoy’s 5A media supplemented with 10% foetal bovine serum and penicillin/streptomycin in a 37 °C humidified incubator with 5% CO_2_ atmosphere. The cells were treated with 25 μM FAM-labelled stapled peptides for four hours before fixation by 3.7% formaldehyde. Nuclei were stained with DAPI-containing mounting media (Olink Bioscience) prior to microscopy. Micrographs were obtained with a Zeiss LSM 510 scanning laser confocal microscope with 63 × /1.4 oil objective.

### Data analysis

The signal intensity of each protein band on the Western blots were normalised with the signal intensity at 37 °C (Supplementary Figure 3). The relative signal intensities were plotted against temperature using Graphpad (Prism) and a variable slope sigmoidal curve was fitted by least squares for each data set using the following equation (1):
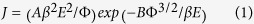


Initial values for top and bottom plateaus were set at 1.0 and 0 respectively for curve fitting, while initial values for Tm50 and Hill Slope were automatically chosen. As the asymmetry of errors is small, errors were assumed to be symmetrical for statistical analyses. Thermal shifts were calculated using ΔT_m_50, which is the difference in temperatures between the control and treatment, at which 50% signal intensity (protein denaturation) was observed.

## Results

### Stapled peptides bind to Mdm2 and Mdm4 at high affinity in CETSA

Stapled peptides based on the N-terminal Mdm2-binding domain on p53 have been shown to successfully inhibit Mdm2 and Mdm4 interacting with p53 in *in vitro* thermofluor assays, as well function in cell based p53 reporter based activation assays. Moreover, similar to the small molecule Mdm2 antagonist nutlin-3, these inhibitors elicit cell cycle arrest and cell death responses in cellular assays from previous studies^9^,^10^,^11^. Because Mdm2/Mdm4 antagonistic stapled peptides are well-studied despite being relatively new, and represent a new potential drug class, they are ideal molecules to test the CETSA method’s ability to assay for affinities, cellular uptake and target engagement *in vivo*. The CETSA data would be invaluable in determining the type of optimisations required to improve the various properties to the stapled peptides, allowing them to become effective therapeutic agents against cancers with wild type p53.

The stapled peptides used in this study have previously been reported to bind strongly to Mdm2 and Mdm4. Table 1 summarises their amino acid sequence, together with their respective IC_50_s reported in the literature. Also included is the IC_50_s for nutlin-3^6^, which will be used as a positive control for Mdm2 thermal stabilisation. These stapled peptides have been shown to specifically inhibit the interaction between p53 and Mdm2/Mdm4^10^,^11^. It is important to note that though the derivation of IC_50_s differ among studies, the implication remains that these compounds exhibit high affinities to Mdm2 and/or Mdm4. The physiological effects of these peptides in activating p53 were recapitulated using a p53 reporter assay (Fig. 2a in Brown *et al.*, 2013). Stapled peptides, or nutlin-3, were added to T22 cells, which have been stably transfected with a p53-responsive β-galactosidase construct^23^, and p53 activation was measured by β-galactosidase activity. Cells treated with increasing concentrations of Staplin, Staplin-2 or ATSP-7041 demonstrated correspondingly increasing p53 activity and plateaued off at maxima, while p53 activation by nutlin-3 exhibited a sharp decline at higher concentrations due to the non-specific effects of high concentrations of nutlin-3. These readouts demonstrated that the stapled peptides were able to activate p53 in cellular assays.

**Figure 1:**
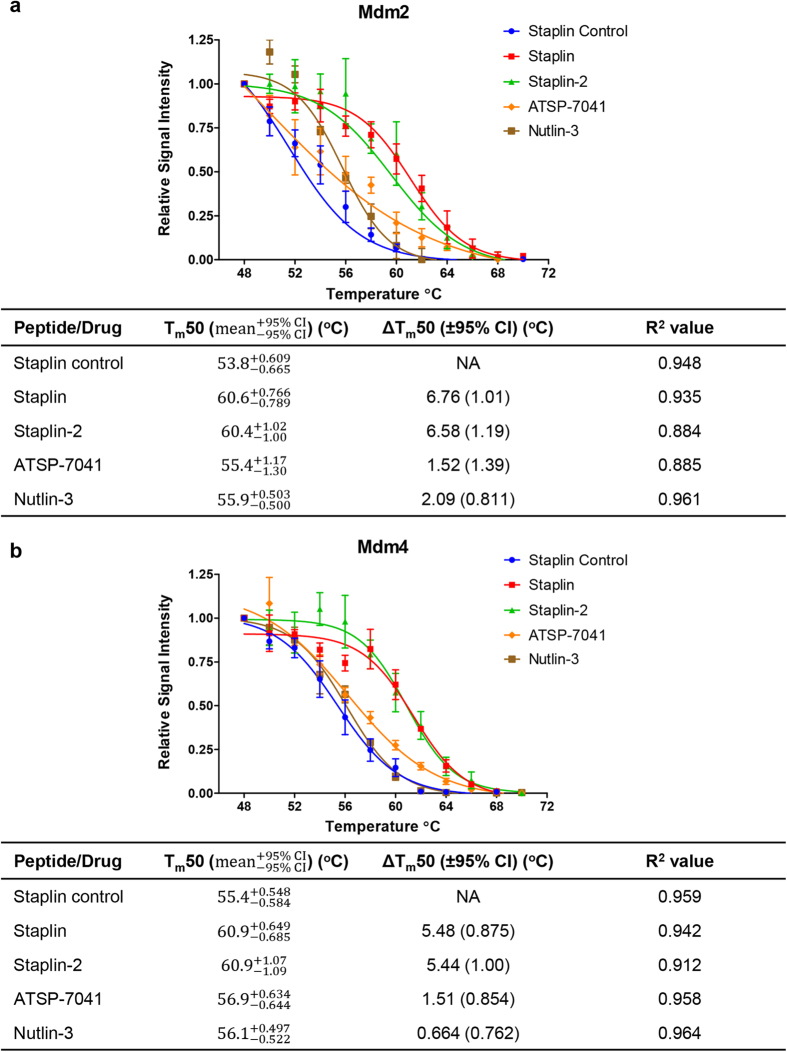
Lysate CETSA demonstrated that stapled peptides based on Mdm2-binding site of p53 bind to both Mdm2 and Mdm4. **a**,**b**. Thermal shift plots of lysate CETSA of nutlin-3 and stapled peptide interactions with Mdm2 (**a**) and Mdm4 (**b**) and their corresponding ΔT_m_50 values with 95% confidence intervals, calculated assuming symmetric errors of T_m_50s. R^2^ values of curve fitting are also included. Nutlin-3 and peptides were added to 5 mg/ml AML2 cell lysates at 10 μM.

The stapled peptides (and nutlin) were analysed for the thermal stabilisation of Mdm2 and Mdm4 in AML2 cell lysates (Fig. 1a,b). Representative Western blots for the stabilisation of Mdm2 are shown in Supplementary Figure 3. Without the addition of Mdm2 specific-antagonists, Mdm2 has a T_m_50 (the temperature at which 50% of proteins are precipitated by thermal denaturation) of 53.8 °C. The addition of nutlin-3 increased its T_m_50 to 55.9 °C. In other words, nutlin-3 resulted in a change of 2.09 °C in T_m_50 over the control, denoted by the ΔT_m_50. In general, the more strongly a ligand bound to a protein, the greater the thermal stabilisation observed^24^. Therefore, the ΔT_m_50 serves as an indication of how strongly the stapled peptides and nutlin-3 is bound to Mdm2, their affinities, in the presence of a multitude of other cellular proteins. In summary, the CETSA results suggest that Staplin and Staplin-2 bound Mdm2 and Mdm4 in cell extracts with high affinities, with ΔT_m_50 of 6.76 °C (Mdm2) and 5.48 °C for (Mdm4) for Staplin and 6.58 °C (Mdm2) and 5.44 °C (Mdm4) for Staplin 2, resulting in the highest thermal shifts in Mdm2 and Mdm4 denaturation in the panel tested, despite a ten-fold smaller IC_50_ reported for nutlin-3. Cellular thermal shifts therefore represent a better indication of efficacy in a cellular environment than IC_50_ values, which only gives an indication of binding affinities in a system containing only purified proteins.

### Assessment of Mdm2/Mdm4 binding by CETSA following modification of stapled peptides

**Figure 2:**
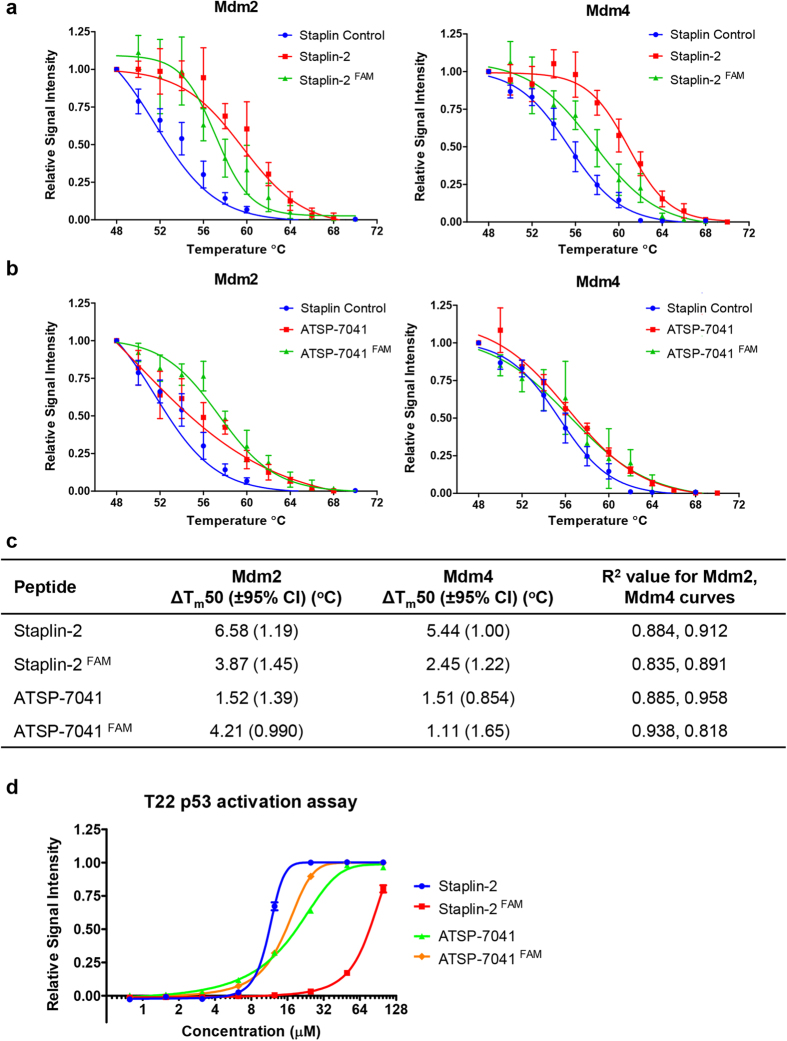
Lysate CETSA can be used to assay for the effect of stapled peptide modification on binding affinities. **a.** Modification of stapling-2 with FAM reduced the binding affinity to Mdm2 and Mdm4. **b.** FAM labelling on ATSP-7041 increased its affinity to Mdm2, but not Mdm4. **c.** Differences in T_m_50 between labelled and unlabelled stapled peptides. **d.** T22 p53 reporter assay with the labelled and unlabelled stapled peptides in the presence of 10% FCS.

As CETSA was developed as a quick way to assay for drugs during optimisation, we were interested in how robust the technique was in assessing target binding following modification of the stapled peptides. FAM (carboxyfluorescein) moieties were conjugated to Staplin-2 and ATSP-7041 and their contribution to Mdm2 and Mdm4 thermal shifts were analysed with CETSA on AML2 cell extracts. Figure 2a depicts the differences between unlabelled and labelled Staplin-2, with the modified Staplin-2 attributing thermal shifts 2.71 °C and 2.99 °C less for Mdm2 and Mdm4 respectively, compared to unlabelled Staplin-2 (Fig. 2c). Interestingly, FAM-labelled ATSP-7041 exhibited increased Mdm2 binding, resulting in a thermal shift of 4.21 °C compared to 1.52 °C of unlabelled ATSP-7041, though its affinity to Mdm4 was weakened, showing a 0.4 °C decrease in thermal shift (Fig. 2b,c). T22 p53 reporter assays using both labelled and unlabelled versions of Staplin-2 and ATSP-7041 showed congruent data, recapitulating the CETSA observations that FAM labelling Staplin-2 negatively affected its activity on Mdm2, while FAM labelling ATSP-7041 enhanced its activity (Fig. 2d). Taken together, these results demonstrated that CETSA can be used to quickly assay for differences in binding affinities during drug modification.

### Whole cell CETSA as a tool to determine cellular entry and target engagement

CETSA can also be used to assay for drug-protein interaction within cells. In this variant, cells were exposed to stapled peptides or nutlin-3 for 4 hours, when cellular uptake of stapled peptides, p53 activity and accumulation of p53 and Mdm2 was observed, but without significant apoptosis or cell death. The treated cells were collected and aliquoted to be heated to increasing temperatures. A simple propidium iodide exclusion assay was performed to determine the maximum permissible temperature of 62 °C before cell membrane failure, well exceeding the temperatures at which thermal stabilisation of Mdm2 and Mdm4 was observed (Supplementary Figure 4).

As the drugs inhibit Mdm2-p53 interaction and therefore degradation, the accumulated p53 would upregulate Mdm2 expression, raising the levels of Mdm2 in the drug-treated cells well above that of the untreated cells. In order to obtain comparable levels of Mdm2 in both the treated and control groups, cells were pre-treated with the proteasome inhibitor MG132 before introducing stapled peptides or nutlin-3 for whole cell CETSAs, or before lysis for cell lysate CETSAs. The accumulation of ubiquitinated proteins may confound the actual denaturation temperatures of Mdm2 and Mdm4, but the addition of proteasome inhibitor is not a variable in this experiment, allowing comparison between techniques and treatment groups.

**Figure 3:**
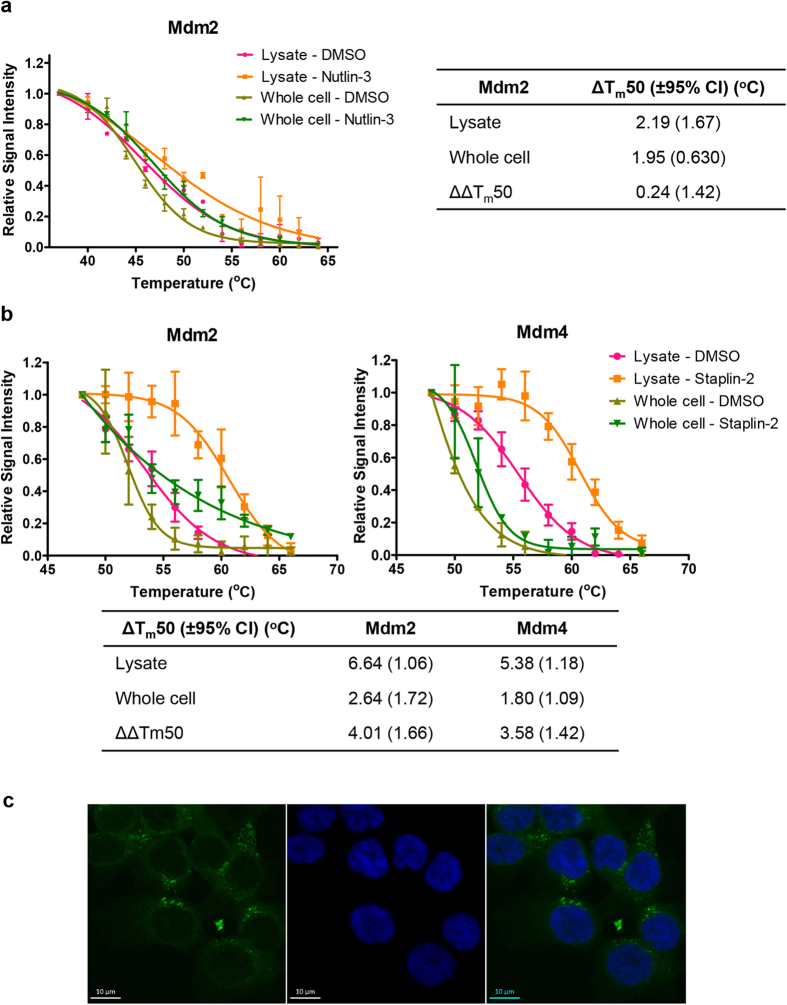
Comparisons between CETSA on whole cells and cell lysates can be used to determine drug cellular entry and target engagement. **a.** The thermal stabilisation of Mdm2 by 10 μM nutlin-3 was assayed by CETSA with cell lysates and whole cells. In both cases, nutlin-3 resulted in very similar thermal shifts, as determined by the ΔΔT_m_50. **b.** The thermal shift of CETSA with whole cells with 25 μM Staplin-2 showed differences in thermal shifts. **c.** Confocal microscopy of HCT116 cells treated with Staplin-2^FAM^ prior to fixation showed vesicular accumulation of the FAM-labelled peptides.

The whole cell CETSA with nutlin-3 produced a thermal shift for Mdm2 that did not differ significantly to that observed in the cell lysate CETSA (Fig. 3a, ΔΔT_m_50 = 0.24 ± 1.42 °C). The mean thermal shift from the whole cell CETSA was 89% of the mean thermal shift for the cell lysate CETSA, indicating that nutlin-3 was able to enter cells and bind to Mdm2 efficiently. On the other hand, despite treatment with a higher peptide concentration, the thermal shifts observed for Mdm2 and Mdm4 for Staplin-2 in the whole cell CETSAs was notably less than recorded for the equivalent cell lysate CETSAs (Fig. 3b, ΔΔT_m_50 = 4.01 °C for Mdm2; ΔΔT_m_50 = 3.58 °C for Mdm4). ). The mean thermal shifts from the whole cell CETSA were 40% and 33% of the mean thermal shift for the cell lysate CETSA for Mdm2 and Mdm4 respectively. This suggests that Staplin-2 cellular entry and target engagement was impaired, and therefore unable to recapitulate the thermal shifts in the cell lysate CETSAs. This is reinforced by the observation of high concentrations of FAM-labelled peptides within vesicles in cells, showing that the availability of stapled peptides for Mdm2/Mdm4 binding was impaired (Fig. 3c).

Comparing the differences in thermal shifts between the cell lysate and the whole cell CETSAs generates an index that correlates with the cellular availability of a drug. The combination of the two assays therefore serve as a powerful tool to enable the optimisation of stapled peptide-based drugs not only in terms of target affinity, but also its ability to enter cells to reach their intended targets.

## Discussion

The efficacy of a drug for therapeutics was commonly assessed by observing for phenotypic changes, such as the shrinking of a tumour mass or lowering of blood pressure. Such an approach to drug discovery led to issues with the activation or inhibition of other cellular pathways, typically resulting in deleterious side effects. This was especially the case for the activation of p53—a multitude of stimuli can drive the activation of p53 and induce downstream phenotypic changes. As our knowledge of biology increases, we came to recognise the importance of designing drugs that target a particular pathway specifically, and drugs designed or discovered to have such properties are, unsurprisingly, better tolerated and generally have better efficacy.

While the concept of stapling peptides is not new, the technology has yet to reach maturity and disparate views have started to appear in the past few years. For instance, the efficacy of Bax-activating stapled peptides has been found to be highly context-dependent, showing enhanced apoptotic activity in some systems but not others^25^,^26^,^27^. Clearly, stapling alone does not enhance a peptide’s efficacy, and until we fully understand the rules for peptide therapeutics, copious optimisation is required to select the ones with the best activities. The process presented in this study aims to provide an effective method to aid in the optimisation of this new class of drugs.

Possibly the most critical way to determine if a drug is specific for a particular pathway is to measure its direct binding to its intended molecular target. Some drugs may be modified to carry affinity tags, which allows direct observations of target engagement though pulldown experiments. However, for many small molecules, the inclusion of an affinity tag adversely affects their binding to their targets, thus indirect measurements of target binding is required.

Several techniques have been developed to measure protein-ligand interactions using spectroscopic and calorimetric analysis^13^,^28^. As described earlier, these methods generally require proteins of very high purity, and in many instances the major bottleneck in drug design and development is the inability to produce purified protein. The CETSA method described by Molina *et al.* was developed to overcome this major obstacle, allow the analysis of protein-ligand interaction in multicomponent solutions—whole cells and tissues, and cell extracts. This method has since been used to validate the binding of several drugs to their protein targets, such as MTH1^29^, Bcl-2/Bcl-xL^30^ and PARP1^31^. Here, we validated the ability of stapled peptide to bind Mdm2 and Mdm4, both in cell extracts, as well as in intact cells.

Besides the main advantage of circumventing the need for purified proteins, CETSA allows the monitoring of drug transport, availability and target engagement within cells. The stapled peptides described in this study have been reported to bind Mdm2 and Mdm4 with very high, low-nanomolar affinities, and this was recapitulated in the cell lysate CETSAs, where massive thermal stabilisation was observed with the addition of these peptides. However, the main disadvantage of these peptides is their relative difficulty in entering cells and reaching their intended targets, as shown by the relatively poor thermal stabilisation in the whole cell CETSA experiments. This may be overcome by altering the staple used to enforce peptide helicity, either by the use of other linkers such as “double-click“ staples^32^, disulphides^33^, thioethers^34^, lactams^35^ or oximes^36^, or by adding functional groups to the alkene hydrocarbon staple to aid in cellular availability. Nonetheless, this study provides strong evidence that CETSA can be employed to investigate drug cellular entry and availability.

The relative ease of the CETSA technique would be invaluable for rapid optimisation of drug design, not just in terms of absolute binding affinities, but also in terms of cellular uptake and target accessibility. The technique is also applicable on samples from animal models and patients^18^. Most importantly, these measurements would be performed on endogenous proteins in their native cellular environments, greatly reducing multiple confounding factors that are associated with similar assays.

## Additional Information

**How to cite this article**: Tan, B. X. *et al.* Assessing the Efficacy of Mdm2/Mdm4-Inhibiting Stapled Peptides Using Cellular Thermal Shift Assays. *Sci. Rep.* **5**, 12116; doi: 10.1038/srep12116 (2015).

## Supplementary Material

Supplementary Information

## Figures and Tables

**Table 1:** Amino acid sequence and their respective IC_50_s (competition assay) of stapled peptides synthesised by Brown *et al*. and Chang *et al*. used in this study.

**Peptide**	**Amino acid sequence**	**Mdm2 IC_50_ (μM)**	**Mdm4 IC_50_ (μM)**
Staplin-Control	Ac-^1^TSAX_r_EYAALAX_s_^11^-NH_2_	>10	>10
Staplin	Ac-^1^TSFX_r_EYWALLX_s_^11^-NH_2_	0.94 ± 0.04^10^	0.66 ± 0.06^10^
Staplin-2	Ac-^1^TSFX_r_EYW(6Cl)WALLX_s_^11^-NH_2_	0.32 ± 0.02^10^	3.49 ± 0.63^10^
ATSP-7041	Ac-^1^LTFX_r_EYWAQ(Cba)X_S_SAA^14^-NH_2_	0.91^11^	2.31^11^
Nutlin-3a	N.A.	0.09^6^	>10^6^

Nutlin-3 was used as a positive control for Mdm2 thermal stabilisation. IC_50_s are obtained from the references indicated.
