# Supplementary material for: Assessing the Efficacy of Mdm2/Mdm4-Inhibiting Stapled Peptides Using Cellular Thermal Shift Assays
Source: Sci Rep. 2015 Jul 10;5:12116. doi: 10.1038/srep12116 (PMC4498326; doi:10.1038/srep12116)
Supplement: Supplementary Information [file srep12116-s1.pdf]

Supplementary Figures for

Assessing the Efficacy of Mdm2/Mdm4-Inhibiting Stapled Peptides Using Cellular Thermal Shift Assays

Ban Xiong Tan[1], Christopher J. Brown[1], Fernando J. Ferrer[1], Tsz Ying Yuen[2], Soo Tng Quah[1], Boon Hong Chan[1], Anna E. Jansson[3], Hsiang Ling Teo[3], Pär Nordlund[3], David P. Lane[1]

[1] p53 Laboratory, A*STAR, Singapore, [2] Institute of Chemical and Engineering Sciences, A*STAR, [3] School of Biological Sciences, Nanyang Technological University, Singapore

Correspondence to: Ban Xiong Tan bxtan@p53lab.a-star.edu.sg
David Lane dplane@p53lab.a-star.edu.sg

Staplin-Control:

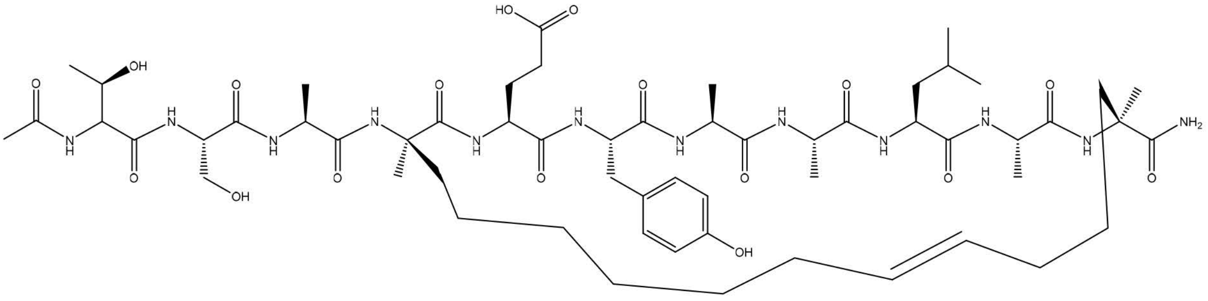

Staplin

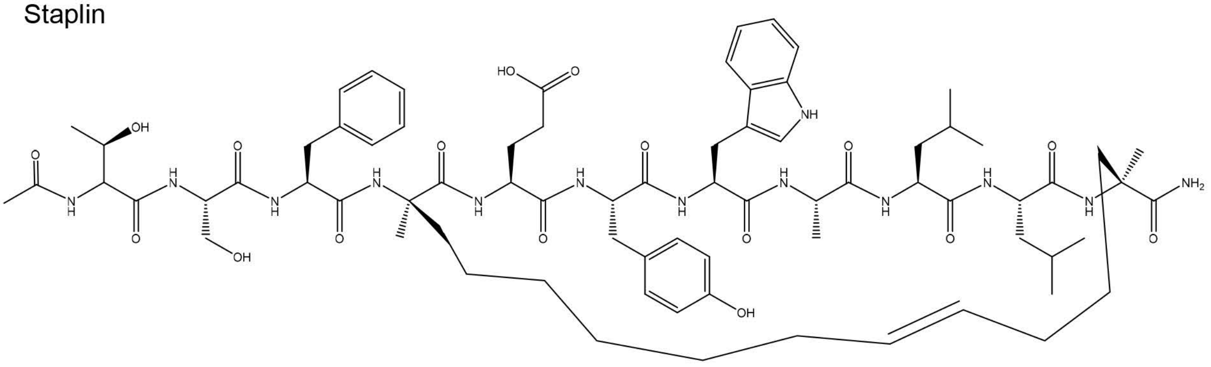

Staplin-2

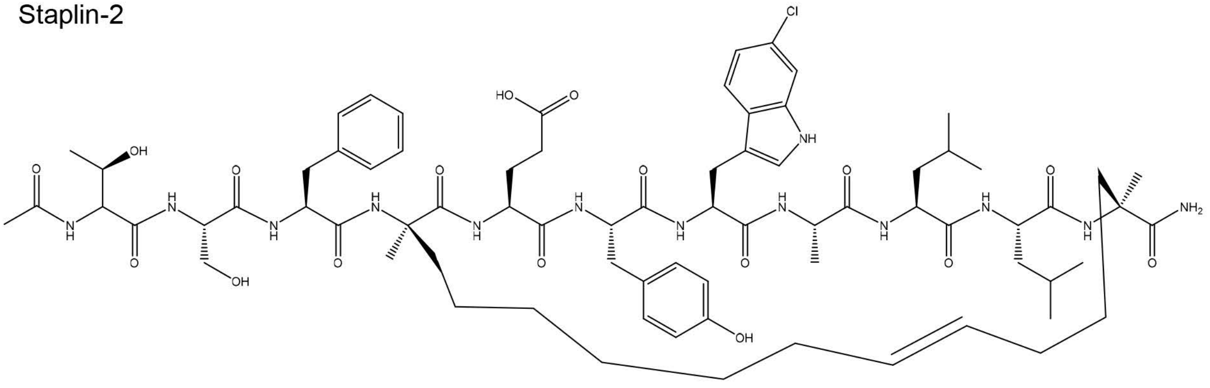

ATSP-7041

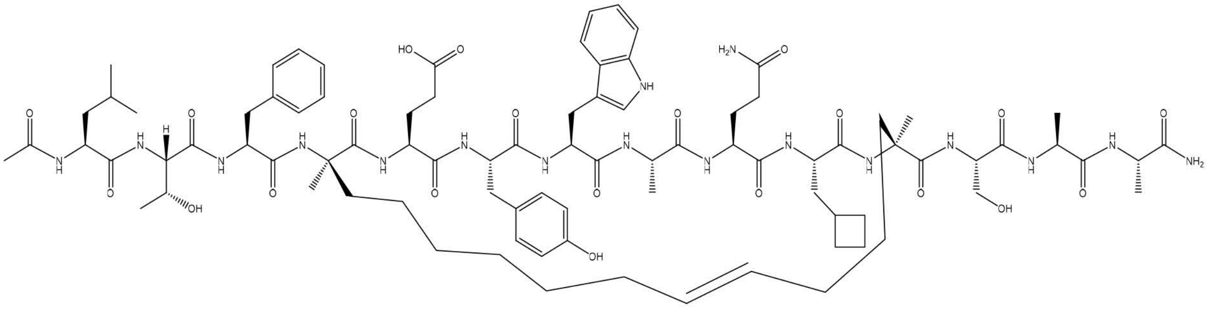

Supplementary Figure 1. Structures of stapled peptides used.
Structures are represented with *trans* hydrocarbon staples, but racemic mixtures were used.

FAM-Staplin-2 (5-FAM with β-alanine spacer)

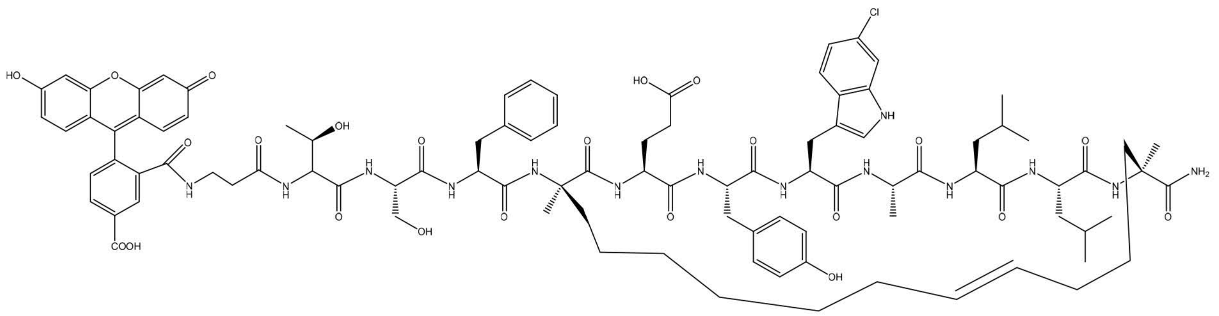

FAM-ATSP-7041 (5-FAM with β-alanine spacer)

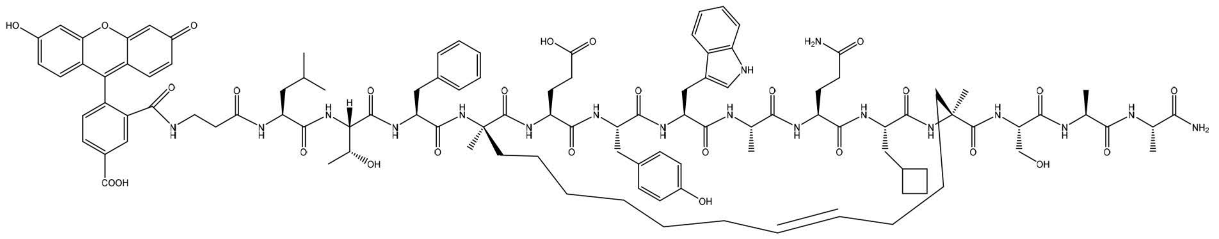

Supplementary Figure 2. Structures of FAM-labelled stapled peptides used.
Structures are represented with *trans* hydrocarbon staples, but racemic mixtures were used.

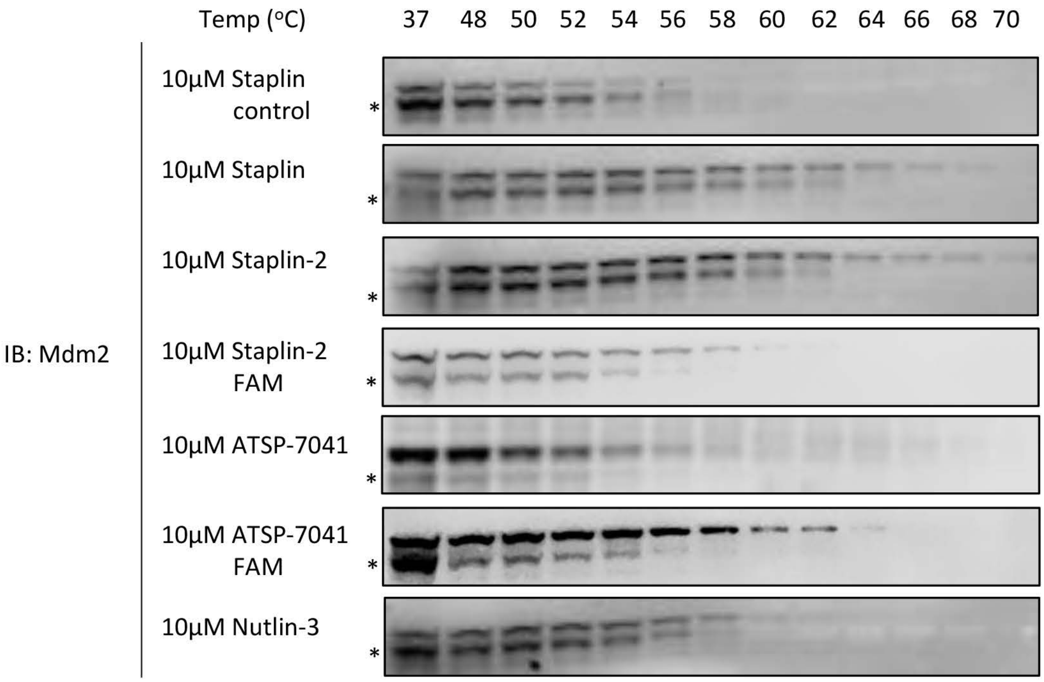

Supplementary Figure 3. Representative immunoblots for thermal stabilisation of Mdm2 with the indicated peptides/compounds.

The compounds were added to 5mg/ml of AML2 cell lysates and heated to the indicated temperatures. Soluble proteins were separated by centrifugation, resolved by SDS PAGE and immunoblotted for Mdm2. Thermal stabilisation curves were plotted using signal intensities of the Mdm2 bands at each temperature point relative to the lowest temperature (37°C).

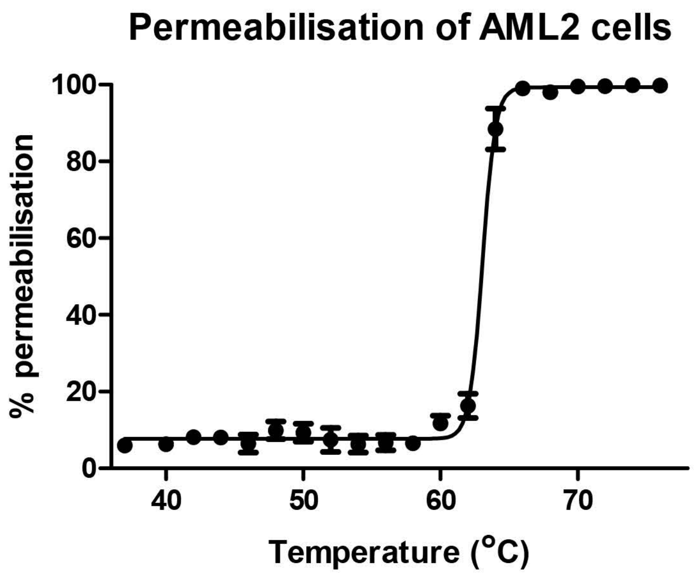

Supplementary Figure 4. Cell membrane failure occurs only at temperatures higher than 62°C.

AML2 cells were subjected to the CETSA protocol by heating with increasing temperatures in the presence of propidium iodide for 2min, then cooled to room temperature for 2min. Cell membrane permeability, assayed by propidium iodide positive cells, was anaylsed by flow cytometry.
